# Third-Party Reproduction - What’s Trending on Instagram?

**DOI:** 10.5935/1518-0557.20230033

**Published:** 2023

**Authors:** Katherine Wede, Alexandra Peyser, Christine Mullin

**Affiliations:** 1 Department of Obstetrics and Gynecology, Division of Reproductive Endocrinology, Northwell Health, North Shore University Hospital, 300 Community Drive, Manhasset, New York, United States

**Keywords:** social media, surrogate, surrogacy, Instagram, reproductive medicine

## Abstract

**Objective:**

The objective of this study was to determine the prevalence, authorship and content type of third-party reproduction-related information shared on Instagram by hashtag analysis.

**Methods:**

A list of 10 hashtags consisting of terms related to third-party reproduction was derived. Content analysis was performed in December 2021 on the most recent 100 posts for each hashtag to determine authorship and content type.

**Results:**

Our search yielded 838,151 posts. The 3 most popular hashtags were ‘surrogacy’, ‘surrogate’ and ‘surrogacy journey.’ Authorship of the top posts were: patients (59.2%), professional society (14.2%), for-profit commercial groups (11.4%), allied health professional (9.4%), physicians (3.3%), and other (2.5%). Patient experiences accounted for the largest share of posts (39.4%), followed by personal posts unrelated to diagnosis (21.5%), outreach posts (19.5%), advertisements (14.2%) and educational (4.8%). Patients authored the majority of posts.

**Conclusions:**

The vast majority of Instagram posts related to third-party reproduction were authored by patients who shared their own personal experiences. Within surrogacy, both gestational carriers and intended parents shared their experiences providing perspective into the surrogacy process. Physician participation may improve the quality and quantity of educational posts and offer a low-cost platform for networking and connecting with patients.

## INTRODUCTION

Over the past 10 years, social media use has grown dramatically (Pew Research, 2021). The public uses social media to connect with others, as well as to gather and share information. It has been reported that 7 in 10 Americans use social media, a 50% increase from 10 years prior (Pew Research, 2021). Instagram, an online platform for photo and video sharing, continues to dominate the online landscape of communication for social media (Pew Research, 2021).

Instagram is a communicative-based social networking service, in which users are able to share and view photos and videos, known as ‘posts’. Users are able to create identification profiles, where their posts are stored. Individuals that post are able to self-categorize their photos and videos into searchable topics that others can find through the use of captions and hashtags. Instagram’s search feature allows users to search and follow particular hashtags and any number of accounts, contributing to the use of Instagram’s self-categorization mechanism. When opening the application, a steady stream of personalized content can be found for each profile, allowing users to become exposed to particular content that is desirable; this content is generated through a user’s following list, but additionally through their activity when searching or simply navigating through the platform. Instagram has successfully developed a community-like feel to their system through the ability to like and follow posts and creators. Users rely upon the like and follow system in order to express gratification toward a particular post or profile, and personalize their home feed. This has allowed for an effective way to initiate and build relationships with those with common interests (Boyd & Ellison, 2007).

Given the rapid changes in the communication landscape brought about by social media use, it is important to establish a better understanding of these technologies and their impact on health communication. Online availability of health-related information has increased significantly following the evolution period of social media platforms (Smailhodzic *et al*., 2016). Though little information is available on the risks of education through social media, it is clear that many users are now utilizing their profiles for the purpose of learning and teaching. Literature has shown that 61% of all American adults have sought health or medical information on the internet, and 49% have accessed a website that provides information about a specific medical condition or problem (Cohen & Adams, 2011). These numbers have increased due to the COVID-19 pandemic (Fullerton, 2021).

Patients, healthcare providers and support groups are now utilizing social media to share their perspective on fertility (Fullerton, 2021). Physicians are able to target patients with interest in the subject matter and patients can search for hashtags that are aligned with their diagnosis. In addition, support groups and professional societies can grow organically through shared hashtags and personalized feeds, which connect similar user content. In addition, patients are also able to share their fertility experience. Previous studies have been performed on hashtag analysis in regard to infertility (Peyser *et al*., 2021). However, to our knowledge, no research has studied third-party reproduction and social media. Therefore, the purposes of this study are: (1) to determine the authorship and types of third-party reproduction-related information targeted toward new patients interested in third-party reproduction on Instagram and (2) to compare the content of posts based on authorship.

## MATERIAL AND METHODS

A list of ten hashtags, correlated to surrogacy, egg donor and sperm donor, were derived, which included: surrogacy, surrogate, surrogate mother, surrogacy journey, surrogacy agency, surrogacy rocks, egg donor, sperm donor, intended parents, and gestational carrier. These hashtags were selected in an attempt to mimic a possible search that a first-time surrogate, intended parent or egg/sperm donor may make. Our methods using Instagram hashtag search and analysis were adapted from previous studies in other fields of medicine (Park *et al*., 2018; Qin *et al*., 2020). An automatic count that is generated by Instagram was utilized in order to record the total number of posts using each hashtag. A total of 100 posts were analyzed for each hashtag, for a total of 1000 posts reviewed. Content analyses were performed to qualitatively evaluate each of the top 100 posts for each hashtag. Clear definitions for each category were designed to minimize any potential bias. Authorship and content type for each hashtag were reviewed on December 30, 2021 by one author (KW). Top posts were determined by Instagram via a proprietary algorithm consisting of parameters, such as the number of likes, comments, and user engagement on the posts.

Content type was divided into the following categories: educational, patient experience, outreach, advertisement, research, personal (unrelated to a diagnosis), and other. Educational posts included posts intended to provide education on third-party reproduction topics. Outreach posts involved posts where the author had been attempting to engage the user. Advertisement-related posts included a clear promotional goal.

Authorship categories included: patient, physician, allied health professional, professional society, for-profit commercial group, and other (law firms, community organizations / non-profit groups, or other professionals not meeting criteria as allied health professionals). This study was reviewed and found to be exempt by the Northwell Health Institutional Review Board.

Posts were excluded that were not in English, were ‘reposts’ of another Instagram account, or were of video form, not image form. Videos were excluded in order to strictly examine differences among posts of photos with captions. All included posts were then assigned to their respective categories for content type and authorship.

## RESULTS

Our search yielded 838,151 posts. The 5 most popular hashtags were surrogacy (n=286,722), surrogate (n=162,090), surrogacy journey (n=76,398), egg donor (n=60,164), and intended parents (n=47,708). Gestational carrier and surrogacy agency had the lowest number of posts (n=39,927 and n=35,404, respectively) (Table 1).

**Table 1:** List of Hashtags.

Hashtag	Number of Posts
Surrogacy	286,722
Surrogate	162, 090
Surrogacy Journey	79, 393
Egg Donor	60,164
Intended Parent	47,708
Surrogate Mother	47,387
Surrogacy Rocks	42,894
Sperm Donor	39,957
Gestation Carrier	39,927
Surrogacy Agency	35,404
Total	838, 651

**Figure 1:**
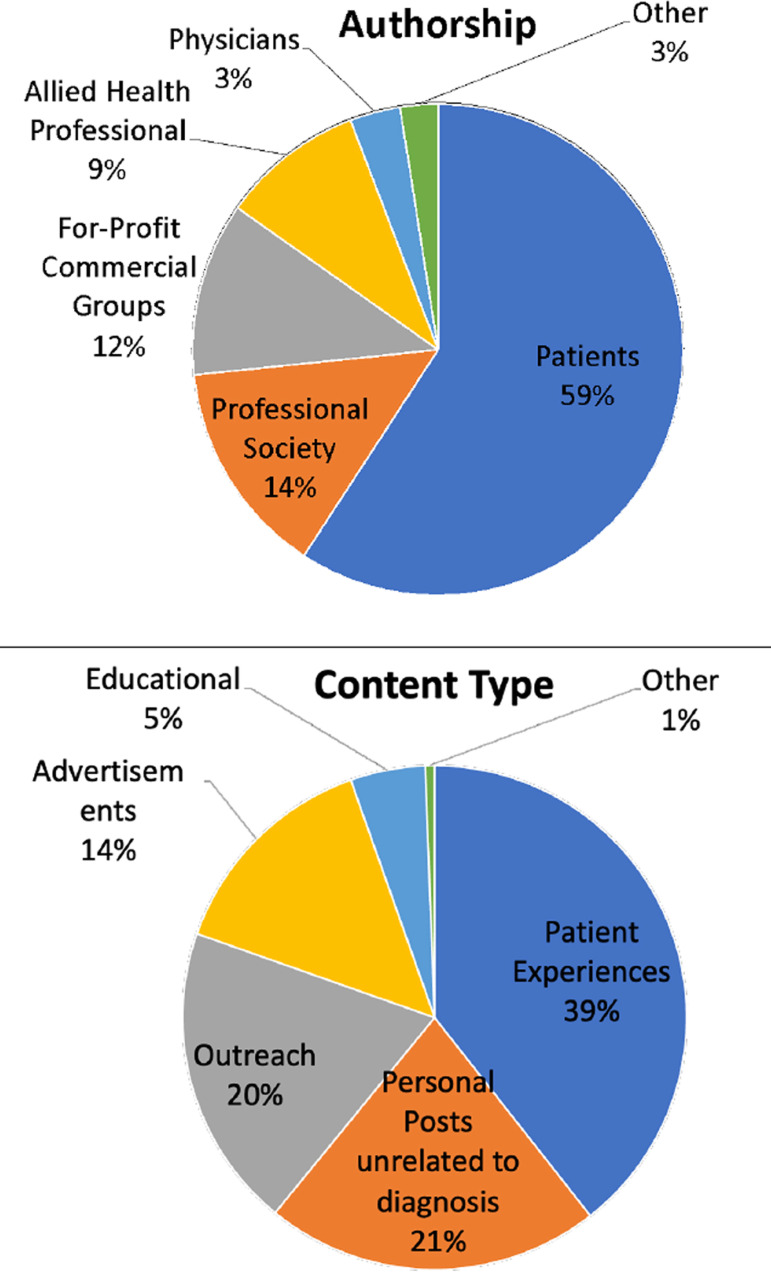
Authorship and content type of third-party reproduction hashtags.

Authorship of the top 100 posts for each hashtag (n=1000) were as follows: patients (59.2%), physicians (3.3%), for-profit commercial groups (11.4%), allied health professionals (9.4%), professional societies (14.2%), and other (2.5%). Of these posts, 39.4% related to patient experiences, 14.2% advertisements, 19.5% outreach, 4.8% educational, and 21.5% personal posts unrelated to diagnoses (Figure 1).

Posts with the hashtag ‘surrogate mother’ and with the hashtag ‘surrogacy rocks’ were authored more by patients than physicians (both 76% *vs*. 2%). ‘Surrogacy’ and ‘surrogate’ hashtags were used in posts that were authored by a significantly lower number of physicians compared to patients (70% *vs*. 0%, and 71% *vs*. 1%, respectively). Authorship of posts with ‘surrogacy agency’ hashtags was dominated by for-profit commercial groups (56/114, 49%). When patients posted about their diagnoses, the hashtags ‘surrogacy rocks’ (64/394, 16%), ‘intended parents’ (68/394, 17%), and ‘gestational carrier’ (69/394, 18%) were used most. Outreach posts tended to contain the hashtag, ‘egg donor’ (64/195, 33%). Authorship by allied health professionals was most prominent in posts with the hashtag ‘surrogacy journey’ (31/94, 33%). Advertisements utilized ‘surrogacy agency’ in 37% (52/142) of posts and ‘surrogacy,’ as well as ‘surrogacy journey’ in 2% (2/94) of posts individually. While 27% of total posts were authored by physicians, allied health professionals, and professional societies, there were no posts related to research and only 5% related to education (Figures 2A, 2B) (Appendix 1).

**Figure 2:**
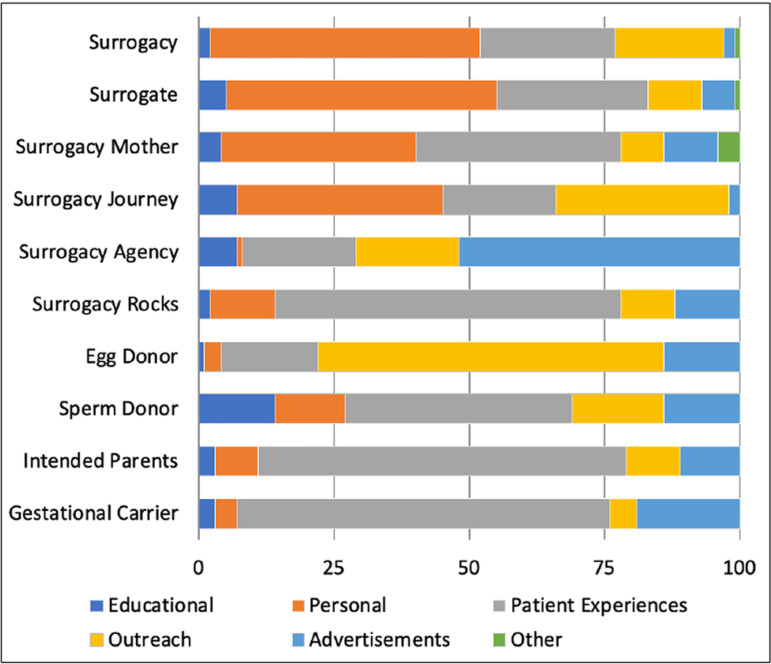
A. Hashtags by content.

**Figure 2:**
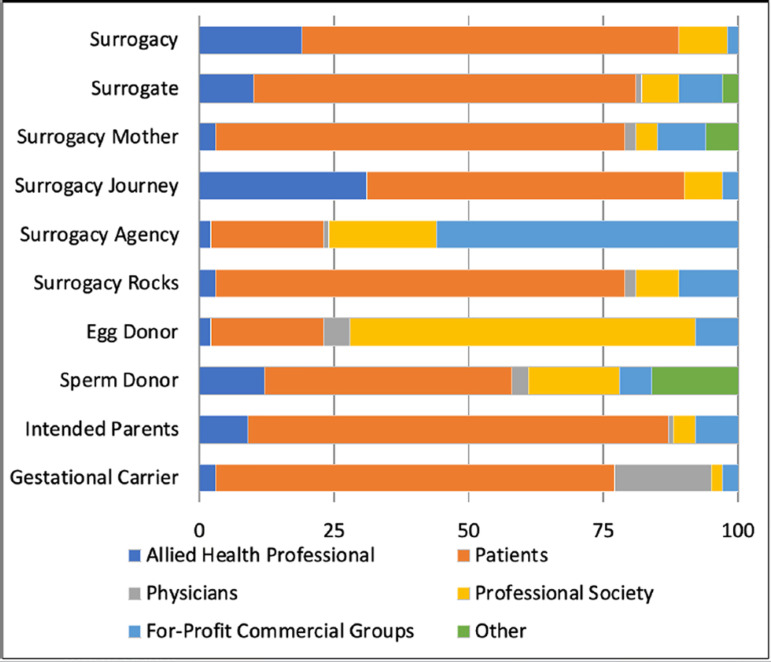
B. Hashtags by authorship.

## DISCUSSION

Our study demonstrates that there is a large amount of third-party reproduction content on Instagram with 59% of posts directed toward patient experience and authored by patients. Only 3% of posts were authored by individual physicians. Fourteen percent of posts were advertisements directed at reproductive-aged women.

The type and content of posts authored by patients compared to other authors significantly differed. Patients were least likely to post about surrogacy agencies and egg donors, whereas others such as for-profit commercial groups and professional societies did so. Patients had a tendency to post with far more personable hashtags demonstrating their journey with treatment. In contrast, for-profit commercial groups and professional societies utilized hashtags directed towards the medical field and surrogacy process in order to connect with clients and future patients or educate the public.

The decision to study Instagram hashtags was determined because it is the feature that connects posts and contributes to its role in developing a sense of community on the platform. Both patients and physicians seek community systems on Instagram for both support and in an effort to educate others within this community. Hashtags allow patients to easily join online communities and engage in conversations about the third-party reproduction process (Douglas *et al*., 2019). Communication devoted to health care topics can be found outside of the clinical setting, which provides a sense of ease and amenity to patients looking to avoid an intimidating visit to an office or hospital. Studies have suggested that online social groups have provided a sense of comfort by reducing social isolation, thereby improving patients’ psychosocial mindset. Patients have reported that they had turned to online health communities in the past to specifically receive empathy through a shared experience of understanding (Nambisan, 2011).

The presence of physicians on social media contributes to the facilitation of evidence-based information-sharing, rather than incorrect opinions or ideas. Though physician presence is important to the accuracy of information found on Instagram, our results have demonstrated that physician presence is heavily lacking with only 3.3% of posts authored by physicians and 14.2% by professional societies. When looking at the content of posts, only a small percentage of posts were considered educational, and there were none that involved research. Therefore, there is a strong need for physicians to enter the Instagram space with the intention of providing evidence-based information to the public via educational or research posts.

Physicians must understand workplace policies surrounding use of social media, the time commitment of posting content, and the implications of participating in discussions online. In addition, many medical societies have developed guidelines on the appropriate use of social media. Both the American College of Obstetricians and Gynecologists (ACOG, 2019) and American Medical Association (AMA) have offered guidance to physicians regarding social media use (AMA). According to a study by Campbell *et al*. (2016), social media use by physicians has been shown to benefit their careers by increasing their reach and audience. Overall, providers have found it desirable and rewarding to connect with communities via Instagram (Campbell *et al*., 2016).

Strengths of our study include its singularity; it is one of the few studies evaluating the third-party reproductive social media webspace, an area that is increasing in utilization. Additionally, the large number of posts analyzed reduces the possibility of randomness within our data. We have identified that patients are interested in describing their experiences with the surrogacy process and the insufficiency of the number of posts authored by physicians. In addition, this study introduces social media to providers who may be unfamiliar with this tool for communication.

Limitations to our study include the use of a single social media platform; results may not reflect that of other social media platforms aside from Instagram. This study developed interpretations and evaluations based on evidence / posts during a limited time frame, while Instagram data is continuously changing. In addition, the chosen hashtags may not be inclusive of all the possible searches someone interested in third-party reproduction may utilize. Some hashtags, though more popular, may have been deemed less likely to be a term one uses upon their first search for more information regarding surrogacy. All possible posts were not viewed because of ‘private’ accounts, making their posts inaccessible. Increasing the time frame studied and number of posts may allow for a more accurate understanding of social media usage by patients and providers.

## CONCLUSIONS

Social media is a communicative platform for the third-party reproduction community that is searching to engage and inform. Patients have an opportunity to gain support and knowledge, and physicians have an opportunity to connect with and educate a large number of individuals through evidence-based posts on social media.

## Appendix

**Appendix 1:** Total posts were.

Hashtag	Surrogacy	Surrogate	Surrogate Mother	Surrogacy Journey	Surrogacy Agency	Surrogacy Rocks	Egg Donor	Sperm Donor
Total Posts	286,722	162,090	47,387	76,398	35,404	42894	60164	39957
Type: Educational Personal posts unrelated to their diagnoses Patient experiences pertaining to their diagnoses Outreach posts Advertisements Research Other **Total**	2 50 25 20 2 -- 1 **100**	5 50 28 10 6 -- 1 **100**	4 36 38 8 10 -- 4 **100**	7 38 21 32 2 -- -- **100**	7 1 21 19 52 -- -- **100**	2 12 64 10 12 -- -- **100**	1 3 18 64 14 -- 0 **100**	14 13 42 17 14 -- 0 **100**
Author: Allied Health Professional Patients Physicians Professional society For-Profit Commercial Groups Other **Total**	19 70 -- 9 2 -- **100**	10 71 1 7 8 3 **100**	3 76 2 4 9 6 **100**	31 59 -- 7 3 -- **100**	2 21 1 20 56 -- **100**	3 76 2 8 11 0 **100**	2 21 5 64 8 0 **100**	12 46 3 17 6 16 **100**
